# Strain Engineering and Halogen Compensation of Buried Interface in Polycrystalline Halide Perovskites

**DOI:** 10.34133/research.0309

**Published:** 2024-02-22

**Authors:** Bin Zhou, Chuanzhen Shang, Chenyun Wang, Duo Qu, Jingyuan Qiao, Xinyue Zhang, Wenying Zhao, Ruilin Han, Shuxin Dong, Yuhe Xue, You Ke, Fengjun Ye, Xiaoyu Yang, Yongguang Tu, Wei Huang

**Affiliations:** ^1^ Frontiers Science Center for Flexible Electronics (FSCFE), Xi’an Institute of Flexible Electronics (IFE) & Xi’an Institute of Biomedical Materials and Engineering (IBME), Northwestern Polytechnical University, Xi’an, Shaanxi 710072, China.; ^2^ Key Laboratory of Flexible Electronics of Zhejiang Province, Ningbo Institute of Northwestern Polytechnical University, 218 Qingyi Road, Ningbo 315103, China.; ^3^ Honors College, Northwestern Polytechnical University, Xi’an 710072, Shaanxi, China.; ^4^ Queen Mary University of London Engineering School, Northwestern Polytechnical University, Xi’an, Shaanxi 710072, China.; ^5^ Key Laboratory of Flexible Electronics (KLoFE) and Institution of Advanced Materials (IAM), Jiangsu National Synergetic Innovation Center for Advanced Materials (SICAM), NanjingTech University, Nanjing, Jiangsu 211816, China.; ^6^ Key Laboratory for Organic Electronics and Information Displays (KLOEID) and Institute of Advanced Materials (IAM), Nanjing University of Posts and Telecommunications, Nanjing, Jiangsu 210023, China.; ^7^ Beijing Solarverse Optoelectronic Technology Co. Ltd, Beijing 100176, China.; ^8^ Intelligent Display Research Institute, Leyard Optoelectronic Co. Ltd, Beijing 100091, China.

## Abstract

Inverted perovskite solar cells based on weakly polarized hole-transporting layers suffer from the problem of polarity mismatch with the perovskite precursor solution, resulting in a nonideal wetting surface. In addition to the bottom-up growth of the polycrystalline halide perovskite, this will inevitably worse the effects of residual strain and heterogeneity at the buried interface on the interfacial carrier transport and localized compositional deficiency. Here, we propose a multifunctional hybrid pre-embedding strategy to improve substrate wettability and address unfavorable strain and heterogeneities. By exposing the buried interface, it was found that the residual strain of the perovskite films was markedly reduced because of the presence of organic polyelectrolyte and imidazolium salt, which not only realized the halogen compensation and the coordination of Pb^2+^ but also the buried interface morphology and defect recombination that were well regulated. Benefitting from the above advantages, the power conversion efficiency of the targeted inverted devices with a bandgap of 1.62 eV was 21.93% and outstanding intrinsic stability. In addition, this coembedding strategy can be extended to devices with a bandgap of 1.55 eV, and the champion device achieved a power conversion efficiency of 23.74%. In addition, the optimized perovskite solar cells retained 91% of their initial efficiency (960 h) when exposed to an ambient relative humidity of 20%, with a T80 of 680 h under heating aging at 65 °C, exhibiting elevated durability.

## Introduction

Metal halide perovskites have attracted considerable attention in the fields of photovoltaics due to their remarkable intrinsic optoelectronic properties, such as tunable bandgap [1], low exciton dissociation energy [2], bipolar transport [3], long carrier diffusion length [4], and well-tolerated defects limit [5]. High-quality perovskite films are a prerequisite for obtaining the high-efficiency perovskite solar cells (PSCs) [6,7]. As is well known, PSCs are mainly based on polycrystalline perovskite films. A great number of defects and imperfections will unavoidably appear in the solution preparation process and will be distributed at the surface and grain boundaries of polycrystalline perovskite thin films. Nowadays, inverted PSCs based on carbazole-based self-assembled monolayers including [2-(9*H*-carbazol-9-yl)ethyl]phosphonic acid, [2-(3,6-dimethoxy-9*H*-carbazol-9-yl)ethyl]phosphonic acid, [4-(3,6-dimethyl-9*H*-carbazol-9-yl)butyl]phosphonic acid, and p-type polymers such as poly[bis(4-phenyl)(2,4,6-trimethylphenyl)amine] (PTAA), and poly(*N*, *N*′-bis-4-butylphenyl-*N*,*N*′-bisphenyl)benzidine have delivered high efficiency, approaching the efficiency of regular counterparts [8–12].

However, the polarity mismatch between these highly nonpolar/hydrophobic organic hole transport layers (HTLs) in inverted PSCs and the highly polar solvents used for perovskite precursor solution, such as *N*-*N*′-dimethyl formamide (DMF) and dimethylsulfoxide, leads to high contact angles and nonideal wetting issues. In consequence, the uniformity and crystallization of polycrystalline perovskite films and the carrier transport dynamics at the interface will be severely affected on the basis of the bottom-up growth process [13–16]. Presently, posttreatment of the directly exposed top surface of polycrystalline perovskite films could more easily enhance the photoelectric properties and stability of the devices, such as 2-dimensional passivation [17–20] or surface reaction [21] to enable interfacial reconfiguration and to anchor surface atoms [22]. However, the buried interface is more worthy of our attention due to the polarity mismatch issue [23–27], which suffers from high-density defect [28], unfavorable strain [29], and deleterious heterogeneity [30].

In the prevailing view, residual strain (or stress) in perovskite films has been proved to severely affect the carrier transport in perovskite/charge transport layer and device stability, which is associated with lattice shrinkage during film formation (annealing or cooling) and coefficient of thermal expansion mismatch between the film and the substrate [29,31,32]. In addition, defects/cracks caused by strain further provide driving forces for ion migration and diffusion of volatile compounds, causing interfacial heterogeneity of the buried interface and leading to degradation of device performance [33]. While charge transport substrates are considered to be the beginning of perovskite periodic epitaxial growth [30], thereby, heterogeneity of the buried interface inevitably leads to localized component inhomogeneity and ion loss at the buried interface [26,34]. At the same time, ion deficiency and the formation of various vacancies at the buried interface of halide-encapsulated crystals usually promoted ion migration, which, in turn, leads to hysteresis behavior and severe interfacial nonradiative recombination [30,35–37]. However, unfavorable strain and deleterious heterogeneity need to be addressed to construct high-performance devices. Therefore, a multidimensional and efficacious method is desired to construct a favorable buried interface conducive to perovskite crystallinity and interfacial contact.

Here, we embed a binary hybrid system of organic polyelectrolyte (PFN-Br) and imidazolium salts to regulate the polarity mismatch issue. After the modification of the PTAA layer by PFN-Br/IAI (organic polyelectrolyte/imidazole hydroiodide), it was revealed that a unique hydrophilic substrate was formed. The dynamic twisting or stretching of the alkyl chains on the PFN-Br effectively reduced the residual strain of the lattice between the perovskite/substrate. Furthermore, because of the free anion (Cl^−^/Br^−^) ionized by PFN-Br/IAI and the Lewis base group on it, we revealed a collaborative mechanism of halogen compensation and immobilization of uncoordinated Pb^2+^ at the buried interface during perovskite crystallization. Benefited from the improved wettability of PTAA substrate, released residual strain, and suppression of nonradiative recombination at the buried interface, the optimized inverted PSC had a PCE (photovoltaic conversion efficiency) of 21.93% based on perovskite with 1.62-eV bandgap. In addition, this coembedding strategy can be extended to RbCsFAMA-based perovskite films, and the champion device achieved a PCE of 23.74%, which performed remarkable ambient and operational stability, thus providing a simple and effective method to improve the performance of PSCs.

## Results and Discussion

**Fig. 1.**
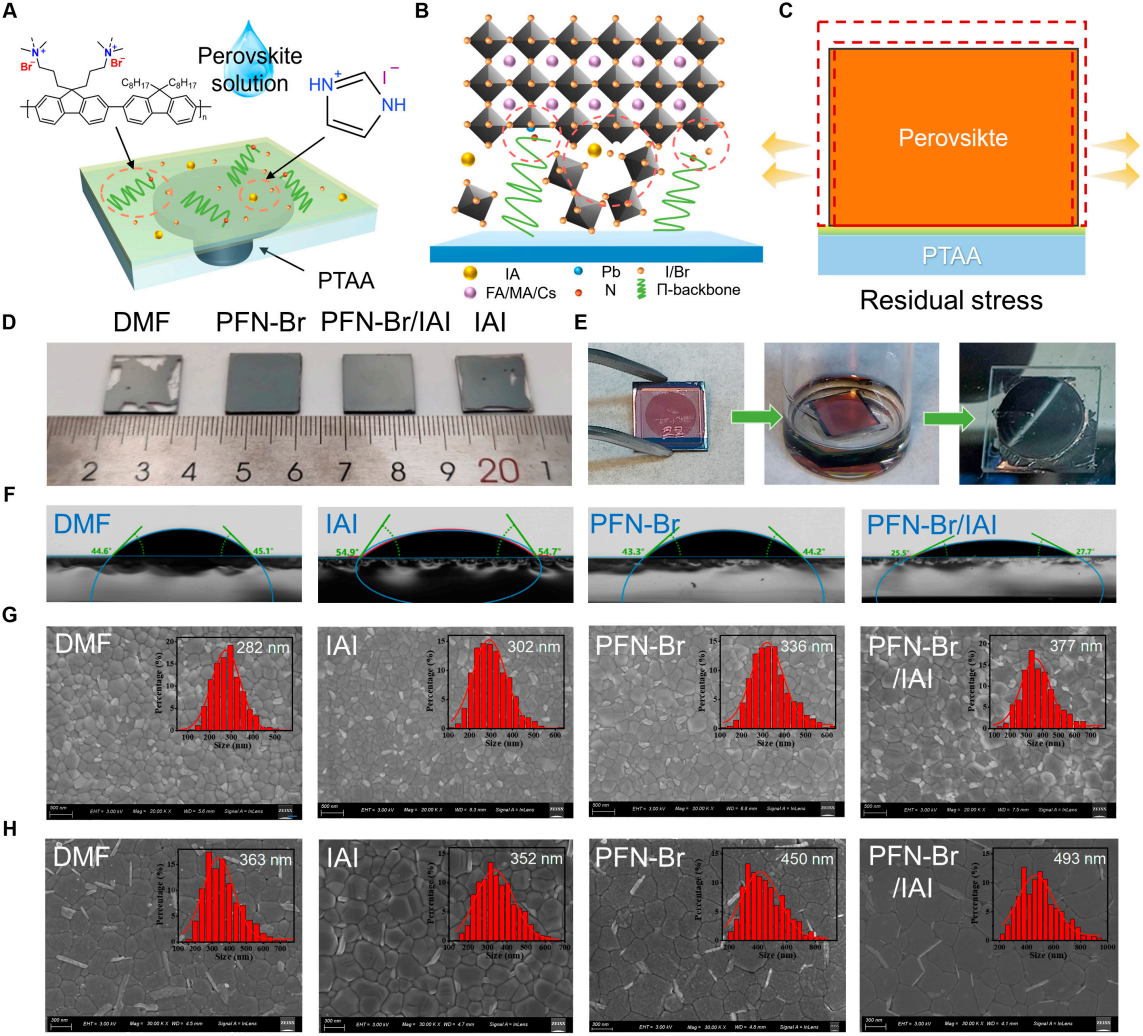
Polarity mismatch modification and the buried interfaces characterization. (A) Schematic of film preparation on the PTAA substrate after modification by the complex (PFN-Br/IAI). (B) Crystallization process of perovskite film. (C) Residual strain relief of perovskite at the modified interface. (D) Photographs of the deposited perovskite films (1.5 cm × 1.5 cm) on PTAA after DMF, PFN-Br, PFN-Br/IAI, and IAI treatments. (E) Uncovering process of simple chemical immersion exposing the buried interfaces. (F) Surface contact angle of perovskite precursor liquid on PTAA after different treatments (here, the residence time is 0 s). (G) Top-view SEM images of perovskite films on different substrates. Scale bars, 500 nm. (H) SEM images of the buried side of the perovskite on different substrates. Scale bars, 300 nm.

Because of the presence of hydrophobic alkyl chains on the polymer hole transport material, it can cause a polarity mismatch problem between PTAA/precursor solution. To achieve uniform and dense perovskite films, PFN-Br, IAI, and PFN-Br with IAI (termed PFN-Br/IAI) were deposited on the top surface of PTAA by spin coating. In addition, PTAA was rinsed with DMF using the conventional method as the control group [38]. In addition, the prepared perovskite films were termed DMF, PFN-Br, IAI, and PFN-Br/IAI groups (Fig. 1A). The perovskite compositions used were based on the (C_S0.05_FA_0.84_MA_0.11_Pb)(I_0.86_Br_0.14_)_3_ (1.62 eV) and (Rb_0.05_Cs_0.05_MA_0.05_FA_0.85_)Pb-(I_0.95_Br_0.05_)_3_ (1.55 eV), respectively (unless otherwise specified, the following performance characterization was mainly based on 1.62-eV bandgap perovskite). Inspired by the unique functionality of PFN-Br [39,40] and IAI (first introduced), we innovatively applied this coembedding strategy to the one-step deposition of perovskite. Such a coembedding strategy is expected to modify the wettability of the substrate and anchor the uncoordinated Pb^2+^ at the buried interface, facilitate telescoping adaptation between the perovskite/substrate, as shown in Fig. 1B and C. (It will be discussed later.)

To investigate the effect of the various pretreatments upon the surface of PTAA, the pretreatment of IAI, PFN-Br, and PFN-Br/IAI seems to increase the coverage of perovskite films, compared to the incompletely covered surface of the DMF group (Fig. 1D). This may be attributed to the higher polarity of the ammonium salts and polyelectrolytes in contact with the precursor solution, which increased the binding force and lowered the contact angle, resulting in full coverage. By dropping precursor solution on the pretreated PTAA substrates (Fig. 1F and Fig. S2), the relevant contact angle of DMF rinse was about 45°, except that IAI increased to about 55° and PFN-Br and PFN-Br/IAI showed a decrease around 44° and 27°, respectively. Thus, the PFN-Br/IAI treatment substantially modified the polarity mismatch between precursor solution and PTAA, facilitating the nucleation growth of perovskite crystals. Curiously, we discovered the presence of island-like distributions on the PTAA after IAI and PFN-Br/IAI treatments (Figs. S3 and S4). On the basis of the energy-dispersive spectrometer mapping analysis of the island structure (Fig. S5), this island structure was introduced by IAI. We hypothesize that the presence of these island-like distributions, which resembles the “papillae” on the surface of the lotus leaf, contributes to the dewetting phenomenon of the IAI-treated PTAA. Besides, since PFN-Br with hydrophilic ionic functional groups [40] in the hybrid system is distributed between the island structures, an excellent wetting surface was formed.

As shown in Fig. 1G and Fig. S6, the top of perovskite films had some smaller PbX_2_ bulges at the grain boundaries [41,42], which is caused by the addition of excessive lead iodide into the precursor. The average perovskite grain sizes were elevated from 302 nm (DMF) to 336 nm (PFN-Br) and 377 nm (PFN-Br/IAI). To further reveal the specific morphology of the buried side of the perovskite layer, we used an updated chemical immersion method (Fig. 1E and Fig. S1) [26]. On the basis of the observation in Fig. 1H, it was known that larger perovskite grain sizes were observed on the buried side and the presence of bright, large schistose Pb halide granules at the grain boundaries that were almost perpendicular to the substrate [26]. We also noted that the buried grains of low-quality perovskite grown on PTAA/DMF and PFN-Br substrates had obvious fragmented crystals on the surface, the number of schistose Pb halide granules was high, and the DMF group had a few obvious pinholes. By contrast, the grains at the buried of the perovskite layer look denser and “cleaner” by IAI treatment, perhaps due to the island-like aggregates acting as nucleation sites during perovskite crystal growth. Consequently, under the synergistic effect of IAI and PFN-Br, a dense buried morphology with almost no surface fragmented crystals, inhibition of schistose Pb halide growth, and grain scale close to 500 nm is formed, which reduced interfacial heterogeneity and enhanced the crystallization quality of perovskite.

**Fig. 2.**
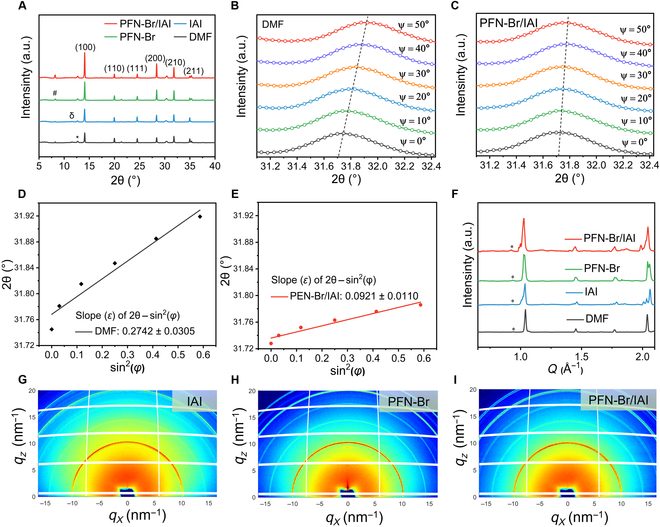
Stress engineering and crystallinity of perovskite films. (A) XRD analysis of perovskite films deposited on different substrates (δ, #, and * represent δ phase, NMP + PbI_2_, and PbI_2_, respectively). The GIXRD spectra of perovskite films of the (B) control and (C) target groups at different Ψ angles (from 0° to 50°). (D and E) Linear fitting of the 2θ − sin^2^ Ψ of the GIXRD spectra. (F) Corresponding radial integral intensity of the following GIWAXS dates. (G to I) 2-Dimensional GIWAXS spectra of the buried side of perovskite films deposited on IAI-, PFN-Br-, and PFN-Br/IAI-treated PTAA layers, respectively (at 0.015° incidence angle). a.u., arbitrary units.

The x-ray diffraction (XRD) spectra used to indeed study the crystallinity of perovskite films on different substrates showed that the peaks of perovskite characteristic crystalline planes, *N*-methyl pyrrolidone (NMP) + PbI_2_ complex phase, δ phase, and PbI_2_, respectively (Fig. 2A). PbI_2_ is the residue of the precursor after the addition of excess Pb iodide, the NMP + PbI_2_ complex phase is the NMP solvent added in the precursor that was not completely volatilized and coordinated with the PbI_2_ during the growth of perovskite. Perovskite films deposited on PFN-Br/IAI showed enhanced peaks at the (100) crystal plane, and the peaks of the nonphotoreactive δ phase were barely visible, indicating that the PFN-Br/IAI-treated substrate promoted the growth of perovskite crystals through the modulation of the polar mismatch and led to the distinct optimization of the crystallinity and the large grains.

Our understanding of the concrete mechanism of such hybrid systems of PFN-Brs and imidazolium salts at the buried interface was still limited. Therefore, in conjunction with the XRD analysis plots (Fig. 2A), we used the Williamson–Hall plot to calculate to semiquantitatively analyze the residual strain of the perovskite crystal films deposited on different substrates (Note S1) [43–45]. As shown in Fig. S7, such compressive strain obtained from the slope of the fitted curves can be attributed to lattice deformation, lattice shrinkage, or preferred orientation of the perovskite during crystallization [31,44]. Compared to the DMF rinse and IAI-treated films, the PFN-Br-treated film achieved a remarkable reduction in residual strain. It was shown that PFN-Br, as a polymer electrolyte that can be twisted and telescopically deformed, can realize the dynamic release of the residual strain of perovskite during the growth and annealing, through the rotation of its alkyl chains [45]. The PFN-Br/IAI-treated films also realized the release of residual compressive strain, which can effectively reduce the defect centers as the lattice strain decreases.

To better avoid misinterpretation and accurately quantitatively evaluate the residual stress (σ) in the perovskite films, we applied the grazing incidence XRD (GIXRD) technique to obtain the GIXRD patterns at different tilt angles from 0° to 50°. As shown in Fig. 2B and C, the characteristic peaks (210) of the control and target films were shifted toward higher angles to different extents, indicating that the lattice gap was reduced, which means compressive residual stress. By fitting a linear relationship of 2*θ* − sin^2^*θ*_0_, the slope *ε* (strain) was found to be remarkably reduced from 0.2742 (control) to 0.0921 after PFN-Br/IAI treatment. According to the following equation [46]:

$$\sigma =-\frac{E}{2\left(1+\nu \right)}\;\frac{\pi }{180^{{}^{\circ}}\;}\text{cot}\theta_{0}\;\frac{\partial \left(2\theta \right)}{\partial {\text{sin}}^{2}\varphi }$$ (1)

where *E* (10.2 GPa) [47] and *ν* (0.31) [33] were the modulus and Poisson’s ratio of the perovskite, respectively, and *θ*_0_ was half of the scattering angle 2*θ*_0_ corresponding to a given diffraction peak of the stress-free perovskite. The results revealed (Table S1) that the *σ* in the control film was about −112.2 MPa; in comparison, the PFN-Br/IAI-based treated film presented a lower stress of about −39.8 MPa. This indicated that the residual compressive stresses of the target films were released to a greater extent (~65% reduction), which improved the substrate/perovskite stretching adaptation to upgrade the quality of perovskite films [29,31], further confirming the analytical results of the Williamson–Hall plot (Fig. S7).

To further exploration of the enhancement of crystallinity on the buried side of perovskite films by strain release, we investigated the buried interfaces by grazing incidence wide-angle x-ray scattering (GIWAXS). As shown in Fig. 2G to I and Fig. S8, it can be seen that *q* = 1.04 A^−1^ and *q* = 0.936 A^−1^ at the buried interface of perovskite, representing the perovskite (100) and the PbI_2_ crystalline planes, and that PFN-Br/IAI treatment had the strongest (100) crystalline plane. According to XRD dates (Fig. 2A), there was no obvious δ phase and NMP + PbI_2_ complex phase in the superficial layer of the buried interface, implying that both of them should be present in the bulk phase or upper surface of perovskite. Although GIWAXS spectra further confirm that the buried interface does not formation of 2-dimensional phases, as shown in Fig. 2F, the coembedding strategy promoted the overall crystallization and growth of perovskite films, which corresponds to the intuitive results of scanning electron microscopy (SEM) (Fig. 1G and H).

**Fig. 3.**
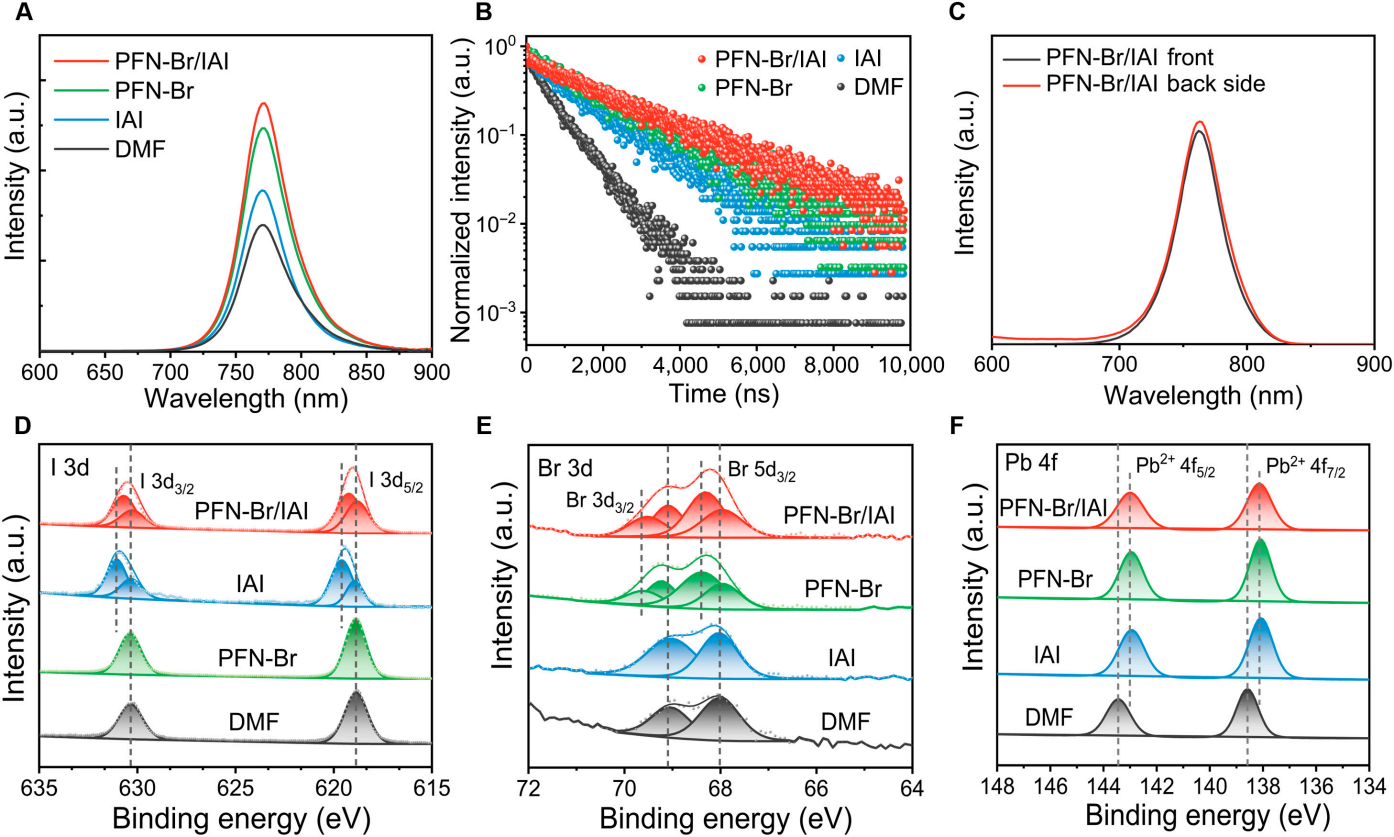
Halogen compensation mechanism. (A and B) Steady-state PL spectra (excitation at 400 nm) and time-resolved PL decay curves (monitored at 780 nm). (C) PL spectra collected from the front and the back of the glass/modified layer/perovskite films. XPS analysis of the buried side of perovskite layers deposited on different substrates concerning the (D) Pb 4f spectrum, (E) I 3d spectrum, and (F) Br 3d spectrum.

The ultraviolet-visible absorption spectra (Fig. S9) showed that the pretreatments on PTAA only enhanced the absorbance in the high-energy spectral region and did not affect the bandgap of perovskite (1.618 eV). To further understand the impact of pretreatment at the buried side on the nonradiative recombination and carrier dynamics of perovskite films, the steady-state photoluminescence (PL) spectrum (Fig. 3A) showed that a considerable enhancement of PL intensity of IAI and PFN-Br groups and the strongest PL intensity was obtained by PFN-Br/IAI. Moreover, the average carrier lifetime (*τ*_avg_) was fitted in the time-resolved PL decay curve (Fig. 3B) using a double exponential function (Table S1 and Note S2). It was found that the *τ*_avg_ of different groups were prolonged from 642.44 ns (DMF) to 2.24 μs (PFN-Br/IAI). The higher PL intensity and longer carrier lifetime suggested that the substantial reduction of traps caused carrier capture. Besides, from the PL signals collected at the front and the back of the perovskite films (Fig. 3C and Fig. S9), the strength of PL on the backside of the pristine film was considerably weaker than on the topside, suggesting a low-quality buried interface, probably the residual strain and halogen deficiency that lead to a large number of trap states. In contrast, the PL strength of the backside of the perovskite deposited on PFN-Br/IAI was even slightly higher than that of the top, which implied that the buried side defects were suppressed to a large extent [28].

To better visualize the chemical interactions on the reduction of defects at the buried side, the exposed buried interfaces were explored using x-ray photoelectron spectroscopy (XPS). According to the XPS spectra of I 3d in Fig. 3D, the characteristic peaks of I 3d exist in the DMF and PFN-Br groups, which were located at 630.34 and 618.86 eV, respectively. Through the peak splitting process, we found the corresponding companion peaks in the IAI and PFN-Br/IAI groups (peaks near 631 and 619.5 eV), which came from the IAI treatment in the shallow surface layer of the buried interface. In addition, the characteristic peak of I 3d in the IAI-treated films shifted toward higher electron binding energies, which was inferred to most likely originate from the filling of iodine vacancy defects at the buried interface with I^−^ in IAI [34,48,49]. Likewise, in the XPS spectra of Br 3d (Fig. 3E), compared with the 2 characteristic peaks of DMF and IAI groups (peaks located near 68.01 and 69.04 eV), the corresponding companion peaks also appeared in the IAI and PFN-Br/IAI treatments (with peaks located near 68.31 and 69.58 eV), and the shifting trend of the Br 3d characteristic peaks were in line with that of I 3d. Defects (halide vacancies) at the interfaces have been known to commonly induce diffusion of halide ions from the perovskite interface into the bulk or the rest of the functional layers, thereby worsening the deleterious interfacial heterogeneity and leading to degradation of device performance [30,35,36]. The appearance of companion peaks indicated that the pretreatments of IAI and PFN-Br brought about changes in the halogen chemical environment, while a higher shift in binding energy indicated an enhanced interaction, which was highly likely to be induced by the compensation of halogen vacancies at the buried interfaces of perovskite with the I^−^/Br^−^ in PFN-Br/IAI.

In addition, in the Pb 4f XPS spectra (Fig. 3F), the DMF-treated perovskite films had 2 peaks located at 138.58 and 143.44 eV, and the binding energy of PFN-Br/IAI-treated film shifted more than 0.44 eV toward lower electron binding energy (peaks at 142.00 and 138.14 eV). This displacement may originate from the fact that the N of PFN-Br and IAI undergoes bonding with the Pb^2+^ due to uncoordinated lone electron pairs. This was further supported by the detection of C–N signals variation in PFN-Br and IAI groups (Fig. S11). The above results demonstrated that the pretreatment of PTAA using the PFN-Br/IAI hybrid system not only released the residual strain in the polycrystalline perovskite films but also formed a beneficial buried interface. Moreover, it compensated for the lack of halogen ions and passivated the undercoordinated Pb^2+^ defects on the buried side, which reduced the possibility of ion migration and the heterogeneity of the buried interface from the source.

**Fig. 4.**
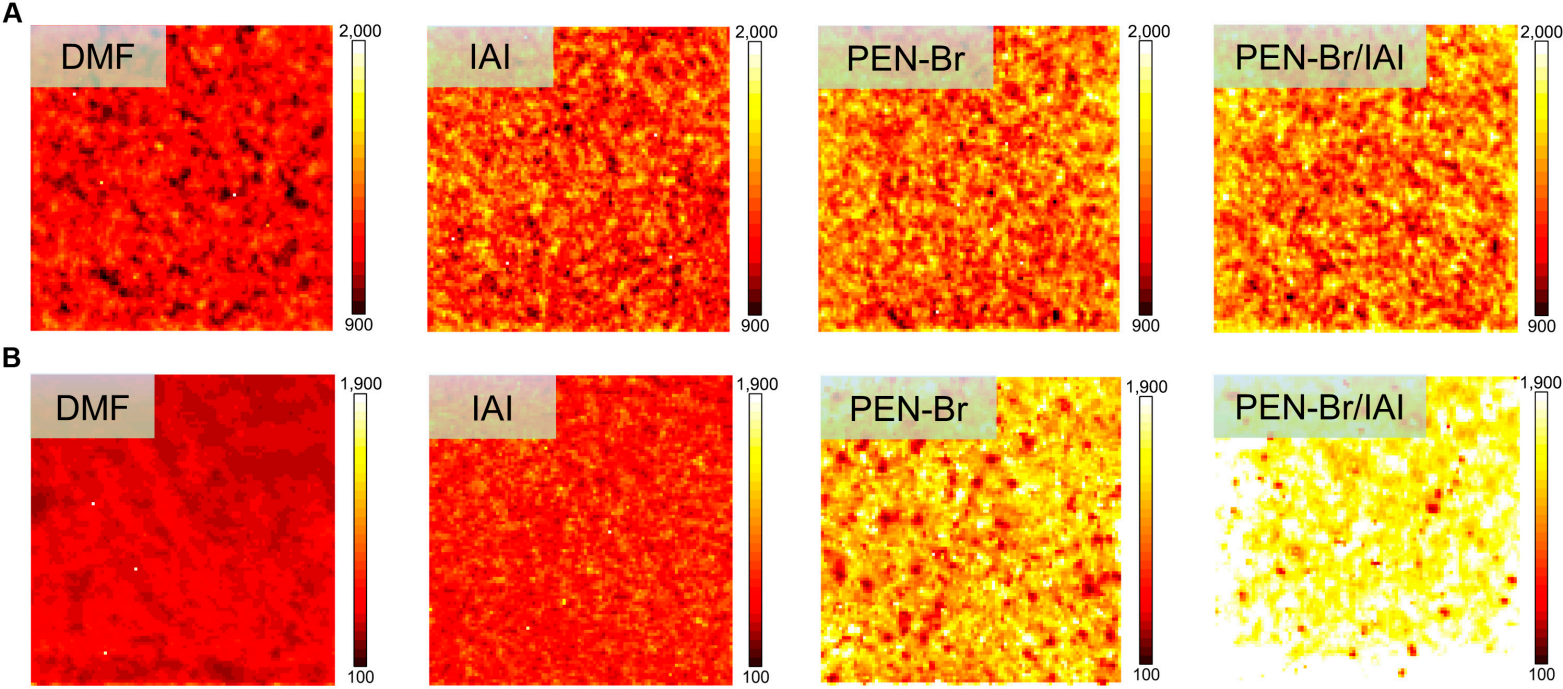
Fluorescence intensity and uniformity at the upper and buried interfaces of perovskite. PL imaging (PL mapping) of the (A) top and (B) exposed buried side of perovskite films deposited on different substrates, respectively.

To visualize the optoelectronic characteristics of the upper and exposed buried interfaces as a whole on a microscopic scale, we used PL imaging (PL mapping) on perovskite films (Fig. 4A and B). Under the same excitation conditions, the trend of PL mapping fluorescence intensity was consistent with that of PL intensity (Fig. 3A), and, remarkably, it seemed that the variation in intensity was more pronounced at the buried side. Therefore, we compared the intensity of the upper and buried sides of the control (DMF) and target (PFN-Br/IAI) groups after normalizing the scale (Fig. S12), and we found that the fluorescence intensity of the buried side of the DMF rinse-treated perovskite remained generally weaker than that of the top, indicating the existence of a great deal of nonradiative recombination regions, which was probably related to the quenching of the buried fluorescence caused by Pb halide [26]. In contrast, the fluorescence intensity of the buried side of the films treated with PFN-Br/IAI was relatively stronger than the top side and more variable than the control. This illustrated that the buried side of the PFN-Br/ IAI sample excites more photons than the upper surface and defects on buried interfaces were substantially reduced, which was consistent with the results of PL spectra (Fig. 3C). In addition, the PL intensity was extracted for the corresponding 10,000 points in the PL mapping (Fig. 4B), showing that the overall PL intensity at the buried side of the hybrid system (PFN-Br/IAI) was higher than that of the rest individual groups (Fig. S12), demonstrating that the nonradiative recombination paths were reduced and the density of nonequilibrium carriers was enhanced.

**Fig. 5.**
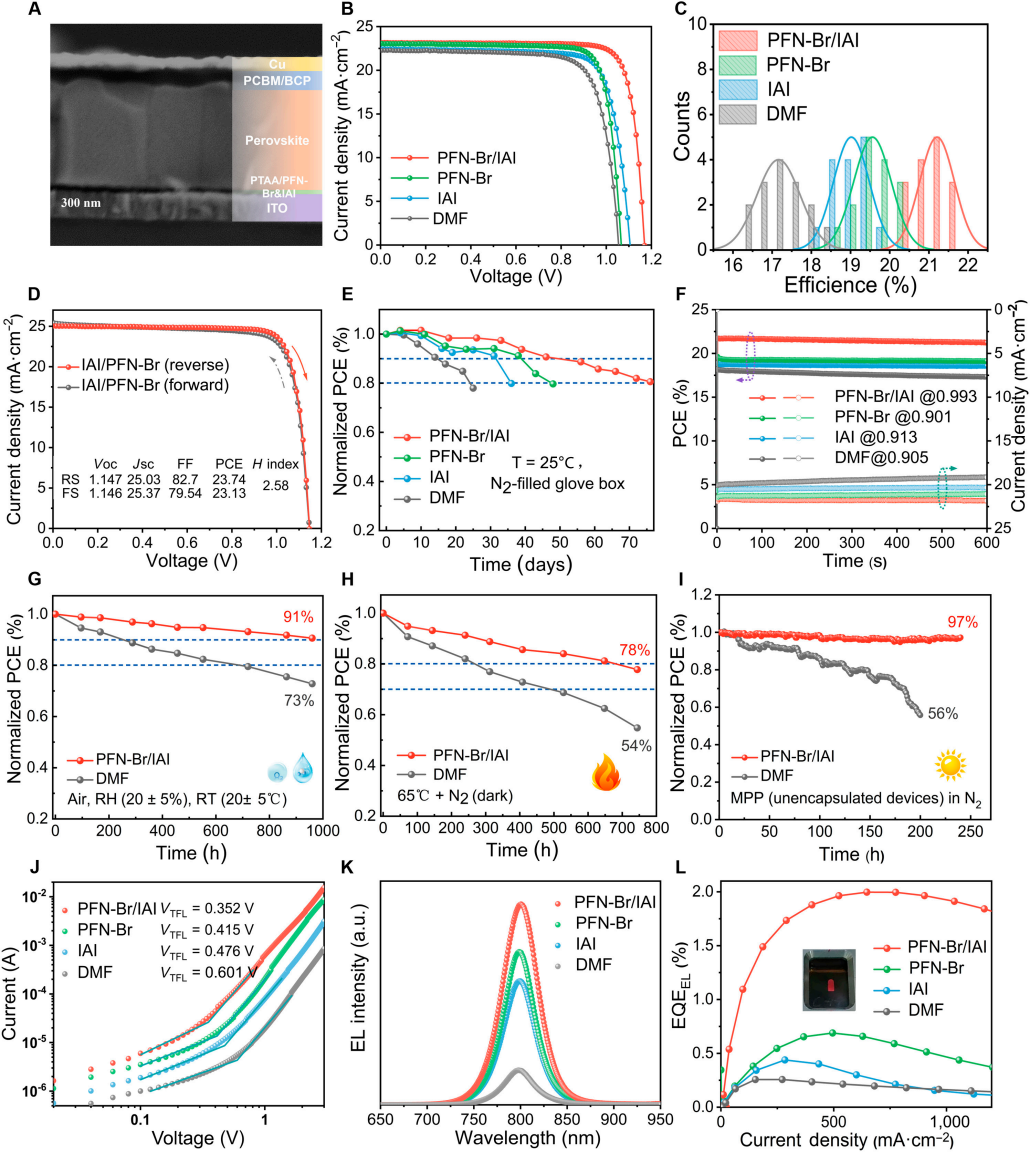
Performance of champion photovoltaic devices processed with PFN-Br/IAI. (A) Cross-sectional SEM image of PFN-Br/IAI-modified PTAA-based invert device. (B) *J*–*V* curves of PSCs based on different modified substrates under simulated standard AM 1.5G illumination (reverse scanning). (C) Statistical PCE distributions of each group of 15 individual PSCs. (D) Forward/reverse scanning *J*–*V* curves and corresponding hysteresis factors (*H* index) of PSCs (1.55 eV) based on PFN-Br/IAI modification substrates. (E) Long-term stability trace for unencapsulated PSCs in N_2_ atmosphere at room temperature (25 °C). (F) Tracking the steady-state power output curves of the PSCs. Normalized PCE evolution of control and target PSCs (G) exposed to an air ambient (aqueous oxygen) and (H) heated at 65 °C in a nitrogen atmosphere under dark condition. (I) Maximum power point (MPP) tracking under continuous light irradiation at 100 mW·cm^−2^ in a nitrogen atmosphere. (J) Estimation of defect density (*N*_t_) of the perovskite film using the HTL-only devices and the corresponding trap filling limit voltage (*V*_TFL_). (K) EL spectra of the PTAA-based PLEDs at a bias voltage of 2.4 V. (L) Plot of the external quantum efficiency versus the injection current density (inset of the target device operated as a PLED).

To evaluate the impact of the hybrid system modified PTAA on the photovoltaic properties of inverted PSCs, a construction of glass/indium tin oxide/PTAA/modification/perovskite/phenyl-C61-butyric acid methyl ester/bathocuproine/copper was prepared (Fig. S13). Compared to the DMF group, the perovskite deposited on PFN-Br/IAI had no obvious pinholes and gaps at the buried side, and the grain boundaries grew vertically, which was believed to be favorable for the longitudinal transport of carriers (Fig. 5A and Fig. S13) [50]. The corresponding photovoltaic performances of PSCs based on different treatments were summarized in Table and Fig. 5B and C. Notably, a satisfactory improvement in the overall PSC performance was realized in the PFN-Br/IAI-treated PSC, with a short-circuit current density (*J*_SC_) of 23.13 mA·cm^−2^, an open circuit voltage (*V*_OC_) of 1.162 V, a fill factor (FF) of 81.60%, and a champion PCE of 21.93%. In contrast, the average efficiency of the target (PFN-Br/IAI) devices is as high as 21.20%, which was remarkably better than the rest of the treated devices in Table. Meanwhile, the hysteresis mainly generated by ion migration was suppressed [51], and the *H* index decreased from 6.1% to 2.0% (Fig. S15). Remarkably, when replacing the photoabsorber with the quadruple-cation perovskite (Rb_0.05_Cs_0.05_MA_0.05_FA_0.85_)Pb(I_0.95_Br_0.05_) (1.55 eV), the corresponding champion device had a PCE of 23.74%, a *V*_OC_ of 1.147 V, an FF of 82.7%, and an *H* index of 2.58 (as shown in Fig. 5D and Table S3). The enhanced device performance can be attributed to the synergistic effects of residual strain relief in the perovskite film and the halogen compensation and Pb coordination at the buried interface after the PFN-Br/IAI pre-embedding treatment. Furthermore, the PCE distribution statistics of PSCs with different pretreatments revealed that the PCE of the PFN-Br/IAI group had a narrow range of variation, indicating better device repeatability and process reliability (Fig. 5C). Consistent with the above analysis of the synergistic mechanism of the hybrid system, the enhanced *J*–*V* characteristics of the devices and the negligible hysteresis largely stem from the improved quality of the perovskite layer, favorable PTAA/perovskite interfacial contact, and suppression of nonradiative composites.

**Table.** Statistics of *J*–*V* parameters of PSCs based on different pretreatments of PTAA (15 cells per group). These data were the mean values and standard deviations of each parameter.

Groups	*V*_OC_ (V)	*J*_SC_ (mA·cm^−2^)	FF (%)	PCE (%)	PCE_max_ (%)
DMF	1.05 ±0.02	22.28 ±0.18	73.26 ±3.02	17.16 ±0.50	18.13
IAI	1.12 ±0.01	22.64 ±0.42	75.32 ±1.83	19.02 ±0.46	19.62
PFN-Br	1.07 ±0.03	22.87 ±0.23	80.19 ±1.32	19.56 ±0.45	20.21
PFN-Br/IAI	1.14±0.02	23.07±0.29	80.72±0.96	21.32 ±0.43	21.93

In Fig. 5E, the normalized PCE intrinsic stability of unencapsulated PSCs was tracked at room temperature (25°C) in the N_2_ atmosphere. It can be seen that the PCE of the DMF, IAI, and PFN-Br groups dropped to about 80% of the initial efficiency after 25, 36, and 48 d, respectively, in contrast to the devices with PFN-Br/IAI treatment, which realized the longest intrinsic stability of 76 d (approximately 1,800 h). It is still mainly originated from the PFN-Br/IAI treatment to form a favorable interface of perovskite/PTAA through the modulation of polar mismatch. Moreover, we attained the steady-state power output curve of the target device with an efficiency of 21.74% at the maximum power point after 600 s of continuous illumination, as shown in Fig. 5F. To assess the impact of external factors on the devices, we performed a series of long-term stability tests on unencapsulated PSCs and films based on the 1.55-eV component (Fig. S16). By tracking the performance evolution of the devices in an air environment [RH (relieve humility), 20 ± 5%; RT (room temperature), 20 ± 5 °C] (Fig. 5G), the target devices based on PFN-Br/IAI maintain more than 91% of the original efficiency after aging for 960 h, compared to only 73% for the control devices, presenting a rather excellent long-term air stability for the target group. The accelerated aging of the perovskite films was carried out by exposing them to air with high humidity (RH, 70 ± 10%), and the variation of their morphology was recorded (Fig. S17). It was found that the control degraded rapidly within 4 d and appeared as a yellow PbI_2_ phase, while the target film appeared to be more resistant. To further evaluate the thermal stability of the device (Fig. 5H), it revealed that the T80 (time required to reduce to 80% of the initial efficiency) of the target PSC under 65 °C heating was over 680 h, but the performance of the control severely degraded to 54% of the original efficiency. As shown in Fig. 5I, the continuous operational stability of the devices was evaluated by tracking the maximum power point under one solar irradiation. Notably, the target device exhibits advanced operational stability, maintaining nearly 97% of the initial efficiency after 240 h of continuous irradiation, while the control device retains only 56% of the initial (200 h). The above results indicated that the released residual stresses and optimized crystallinity in the perovskite films not only improved the device efficiency but also helped to achieve superior long-term stability when the pre-embedded coembedding strategy was used.

To quantitatively assess the defect density (*N*_t_) in perovskite films deposited on different substrates, we fabricated the HTL-only transport devices with indium tin oxide/PTAA/modified layer/perovskite/Spiro-OMeTAD/Au structure. In conjunction with the space charge limiting model, as illustrated in Fig. 5G and Note S3, the corresponding trap filling limit voltage (*V*_TFL_) can be gained [52,53]. When compared with the *V*_TFL_ of 0.601 V of the control device, the *V*_TFL_ of the PFN-Br/IAI-treated device was only 0.352 V. The *N*_t_ of the equivalent HTL-only devices were 5.99 × 10^15^ cm^−3^ and 3.51 ×and^15^ cm^−3^, respectively, demonstrating that the coembedding strategy effectively diminished the trap-state density of the perovskite bulk by 41% and well suppressed the interfacial nonradiative recombination [54]. To further visually gauged the role of defect reduction on the optoelectronic performance of the devices, we used PSCs with different interfacial modifications as perovskite light-emitting diodes (PLEDs) to obtain the external quantum efficiency of electroluminescence (EQE_EL_) during EL at a bias voltage of 2.4 V. As presented in Fig. 5H and I, the PLED based on the PFN-Br/IAI treatment with red light emission exhibited about 2.0% of the best EQE_EL_ and the highest EL intensity. The center of the EL spectrum is at 798 nm, and there is a small shift within the acceptable range to the parameters measured in the PL spectra (Fig. 3A). On the contrary, the much lower EQE_EL_ of 0.257%, 0.439%, and 0.689% for the PLEDs is based on DMF, IAI, and PFN-Br, respectively. It was well known that superior PSCs should be an excellent PLED as well, and, therefore, the EL/EQE_EL_ can reflect the nonradiative recombination of the devices [27]. The defect-assisted recombination and carrier transport losses were key factors in open-circuit voltage losses in PSCs/PLEDs [55]. Hence, the enhanced EL performance would imply a reduction of deep energy level defects within the PSCs, leading to a remarkably higher PCE.

## Conclusion

In summary, we demonstrated that a hybrid system of PFN-Br and imidazolium analog salts (IAI) embedded at the buried interface of perovskite could regulate the polarity mismatch issue and improve the photovoltaic performance of inverted PSCs. After the modification of the PTAA layer by PFN-Br/IAI, the dynamic twisting or stretching of the alkyl chains on the PFN-Br effectively reduces the residual strain of the lattice between the perovskite/substrate. Furthermore, because of the free anion (Cl^−^/Br^−^) ionized by PFN-Br/IAI and the Lewis base groups on it, we revealed a collaborative mechanism of halogen compensation and immobilization of uncoordinated Pb^2+^ at the buried interface during perovskite crystallization. Benefited from the improved wettability of PTAA substrate, residual strain relief, and suppression of heterogeneity at the buried interface, it could facilitate the obtaining of dense and uniform high-quality perovskite film, enhance the carrier electrical transport properties, and reduce the nonradiative recombination. As a consequence, the optimized inverted PSC has a PCE of 21.93% (*V*_OC_ of 1.16 V) based on perovskite with a 1.62-eV bandgap with a reduction of defect state density and remarkable intrinsic stability. In addition, this coembedding strategy can be extended to RbCsFAMA-based perovskite films, and the champion device achieved a PCE of 23.74% (bandgap of 1.55 eV) compared to 21.47% (control) with negligible hysteresis and remarkable resistance to external conditions and operational stability. Thus, this research offered a simple and effective coembedding design strategy that can synergistically enhance device efficiency and endurance.

## Materials and Methods

Detailed Materials and Methods can be found in the Supplementary Materials.

## Data Availability

All data needed to evaluate the conclusions in the paper are present in the paper or the Supplementary Materials.

## Supplementary Materials

Supplementary 1 Materials and Methods Figs. S1 to S17 Tables S1 to S4 Notes S1 to S3
