# Supplementary material for: Strain Engineering and Halogen Compensation of Buried Interface in Polycrystalline Halide Perovskites
Source: Research (Wash D C). 2024 Feb 22;7:0309. doi: 10.34133/research.0309 (PMC10882268; doi:10.34133/research.0309)
Supplement: Supplementary 1 — Materials and Methods Figs. S1 to S17 Tables S1 to S4 Notes S1 to S3 [file research.0309.f1.docx]

**Supplementary Materials**

**Strain engineering and halogen compensation of**

**buried interface in polycrystalline halide perovskites**

Bin Zhou^1^, Chuanzhen Shang^1^, Chenyun Wang^1^, Duo Qu^1^, Jingyuan Qiao^1^, Xinyue Zhang^1^, Wenying Zhao^1^, Ruilin Han^1^, Shuxin Dong^3^, Yuhe Xue^4^, You Ke^1^, Fengjun Ye^7^, Xiaoyu Yang^8^, Yongguang Tu^1,2^* and Wei Huang^1,2,5,6^

^1^Frontiers Science Center for Flexible Electronics (FSCFE), Xi’an Institute of Flexible Electronics (IFE) & Xi’an Institute of Biomedical Materials and Engineering (IBME), Northwestern Polytechnical University, Xi’an, Shaanxi 710072, China. ^2^Key Laboratory of Flexible Electronics of Zhejiang Province, Ningbo Institute of Northwestern Polytechnical University, 218 Qingyi Road, Ningbo 315103, China. ^3^Honors College, Northwestern Polytechnical University, Xi’an 710072, Shaanxi, China. ^4^Queen Mary University of London Engineering School, Northwestern Polytechnical University, Xi’an 710072, Shaanxi, China. ^5^Key Laboratory of Flexible Electronics (KLoFE) and Institution of Advanced Materials (IAM), Jiangsu National Synergetic Innovation Center for Advanced Materials (SICAM), NanjingTech University, Nanjing, Jiangsu 211816, China. ^6^Key Laboratory for Organic Electronics and Information Displays (KLOEID) and Institute of Advanced Materials (IAM), Nanjing University of Posts and Telecommunications, Nanjing, Jiangsu 210023, China. ^7^Beijing Solarverse Optoelectronic Technology Co., Ltd, Beijing 100176, China. ^8^Intelligent Display Research Institute, Leyard Optoelectronic Co., Ltd, Beijing, 100091, China.

*Address correspondence to: iamygtu@nwpu.edu.cn

**Materials and Methods**

**Materials**

PbI_2_ (99.99%), Imidazole Hydroiodide(IAI) (98%), and PbBr_2_ (98%) were purchased from Tokyo Chemical Industry Co., Ltd (TCI). FAI was purchased from Greatcell Solar Co., Ltd (Dyesol). Poly[bis (4-phenyl) (2,4,6-trimethylphenyl)amine] (PTAA, Mw≈12000), bathocuproine (BCP), phenylethylammonium chloride (PEACL), CsI and MABr was purchased from Xi’an Polymer Light Technology Corp. Copper(Cu) was received from commercial sources with the high purity (≥99.99%). [6,6]-phenyl-C61-butyric acid methyl ester (PCBM) and Poly[9,9-bis(3’-(N, N-dimethyl)-Nethylammoinium-propyl-2,7-fluorene)-alt-2,7-(9,9-dioctylfluorene)] dibromide (PFN-Br) was purchased from Solarmer Materials Incorporation. Methanol (99.9%) was purchased from Adamas-beta (China). *N, N*-dimethylformamide (DMF, 99.8%), Dimethyl sulfoxide (DMSO, 99.8%),1-Methyl-2-pyrrolidinone (NMP, 99.5%), acetonitrile (99.9%), chlorobenzene (CB,99.8%), and isopropanol (IPA, 99.5%) were purchased from Acros without further purification.

**Methods**

**The solution preparation:** The 1.3 M perovskite precursor for the perovskite (C_S0.05_FA_0.84_MA_0.11_PbI_2.58_Br_0.42_) was prepared by dissolving the PbI_2_ (529 mg), FAI (187.4 mg), PbBr_2_ (74.3 mg), MABr (15.73 mg) and CsI (17.55 mg) in a mixed solvent of 800 μL DMF, 150 μL DMSO and 50 μL NMP solvents, respectively. Afterward, the solution was heated at 70℃ and stirred for 1 hour before use. The 1.5 M perovskite precursor for the perovskite (Rb_0.05_Cs_0.05_MA_0.05_FA_0.85_)-Pb(I_0.95_Br_0.05_)_3_ was prepared by dissolving the PbI_2_ (656.9 mg), FAI (219.5 mg), PbBr_2_ (27.5 mg), MABr (8.4 mg) , CsI (19.5 mg) and RbI (15.9 mg) in 1 mL DMF:DMSO (v:v=4:1). Afterward, the solution was heated at 60℃ and stirred for 1-2 hour before use. The solution for the hole transporting layer (HTL) was prepared with PTAA diluted in chlorobenzene (2 mg mL^-1^). The solution for the electron transporting layer (ETL) was prepared with PCBM diluted in chlorobenzene (20 mg/mL). The solution for the buffer layer was prepared with BCP dissolved in IPA (0.5 mg/mL). The solution for the post-treatment was prepared with PEACL (1.0 mg/mL in IPA). The solution for the buried modified layer was prepared with PFN-Br dissolved in methanol (0.5 mg mL^-1^), and IAI dissolved in DMF (4 mg mL^-1^). For the preparation of PFN-Br/IAI solution, the PFN-Br/IAI with a weight ratio of 1:4 and a total concentration of 1.25 mg mL^−1^ was dissolved in methanol, and the solution was stirred at 50℃ for 20 min before use. The complete process was carried out in the nitrogen-filled glove box.

**Solar Cells Fabrication**: The indium tin oxide (ITO) transparent conductive substrates were cleaned through sequential ultrasonication in deionized water, detergent, acetone, and isopropanol for 20 min each. Before use, the ITO substrates were dealt with UV-ozone for 20 min. For the fabrication of inverted PSCs with the one-step method, the PTAA was spin-coated (6000 rpm, 30 s) onto the ITO glass substrate, and then annealed at 100℃ for 10 min. After the films cooled down to room temperature, for the sole DMF rinse treatment, the DMF (100 μL) was spin-coated upon the PTAA layer at 4000 rpm for 10 s. For the sole PFN-Br treatment, the PFN-Br solution was dynamically spin-coated upon the PTAA layer at 5000 rpm for 30 s. As for sole IAI treatment, the IAI solution was spin-coated upon the PTAA layer at 5000 rpm for 30 s, and then heated at 100℃ for 5 min. For the PFN-Br/IAI treatment, the hybrid solution was spin-coated upon the PTAA layer at 5000 rpm for 30 s, followed by 100℃ annealing for 5 min. When the substrate was cooled to room temperature, the subsequent perovskite film was deposited on the modified PTAA substrate. The perovskite precursor solution (1.3 M) was spin-coated on a modified PTAA layer by a two-consecutive step program at 200 rpm/s, 2000 rpm for 10 s, and 1000 rpm/s, 6000 rpm for 30 s, respectively. During the second step, 150 μL of CB was poured on the center of the spinning substrates 16 s before the end of the whole spinning program. Subsequently, the films were annealed at 100°C for 60 min. The perovskite precursor solution (1.5 M) was spin-coated on a modified PTAA layer by a two-consecutive step program at 200 rpm/s, 1000 rpm for 10 s, and 2000 rpm/s, 3000 rpm for 40 s, respectively. During the second step, 150 μL of CB was poured on the center of the spinning substrates 25 s before the end of the whole spinning program. Subsequently, the films were annealed at 100°C for 10 min. After the perovskite was cooled for 10 min, the perovskite films were treated by PEACL under 2500 rpm/s, 5000 rpm for 30 s, and annealed at 100℃ for 10 min. PCBM was spin-coated on perovskite later with a speed of 1200 rpm/s for 30 s. BCP was then spin-coated on PCBM at 5000 rpm/s for 30s. Finally, a 100 nm copper electrode was thermally evaporated in a vacuum chamber with a shadow mask, making sure that the active working area of PSCs is 0.062 cm^2^. For devices based on PTAA/DMF, PTAA/PFN-Br, PTAA/IAI, and PTAA/PFN-Br&IAI, the fabrication process of each layer is the same as mentioned above.

**Film Characterization**

Surface morphology of PTAA after different pretreatments observed by optical microscopy(Olympus, BX53M). The UV-vis absorption spectra of perovskite films were measured by a spectrophotometer (UH4150, Hitachi). Surface micro- and nanoscale morphologies were obtained through field-emission scanning electron microscopy (FESEM, Gemini 300). The crystalline structures were characterized by X-ray diffraction (XRD, Bruker AXS, D8 Advance). The steady-state fluorescence spectrum and time-resolved PL spectra were acquired using the FLS1000 Photoluminescence Spectrometer. The confocal photoluminescence mapping images were measured with the laser scanning confocal microscope (Enlitech, SPCM-1000), it was equipped with a 532 nm laser, and the power was 5 μW. The hydrophilicity of surfaces as a function of time was measured by using a drop shape analyzer (KRÜSS, DSA100) at ambient temperature. The surface electronic states were measured by ultraviolet photoelectron spectroscopy (UPS, Axis Ultra, Kratos Analytical Ltd). Grazing-incidence wide-angle X-ray scattering (GIWAXS) measurements were performed at the BL17B1 beamline of Shanghai Synchrotron Radiation Facility (SSRF), Shanghai, China, using beam energy of 10 keV (λ = 1.24 Å) and a Pilatus 3S-2M detector. Note that the incidence X-ray beam was adjusted to 0.15 degree for detecting the crystallographic information in the shallow depth of the film. The above relates to the characterization of the buried bottom of perovskite, e.g., PL, PL-mapping, SEM, XPS, GIWAXS, all the samples were fabricated on the pre-processed ITO/PTAA substrates, and then the structure of Glass/UV curing glue/copper/perovskite was obtained after chemical immersion stripping.

**Solar Cell Characterizations**

The current density-voltage (*J-V*) curves of solar cells were measured (2400 Series SourceMeter, Keithley Instruments) under standard simulated AM 1.5 G sunlight at 100 mW·cm^-2^ irradiance by a 150 W class AAA solar simulator (XES-40S1, SAN-EI). The light intensity of 100 mW·cm^-2^ was calibrated using a standard monocrystalline silicon solar cell with a KG-5 filter. The measuring condition was reverse scan (1.22 V → -0.02 V, scan rate 40 mV/s, and no delay time) and forward scan (-0.02 V → 1.22 V, scan rate 40 mV/s, and no delay time). The *J-V* measurements were performed in an N_2_ environment without encapsulation. Stabilized power output (SPO) and time-dependent stability trace of unencapsulated PSCs were also measured in an N_2_ environment under simulated AM 1.5G, 100 mW cm^-2^ solar irradiation, at room temperature (25°C). The cells were masked to give the defined active area of 0.062 cm^2^. For the external quantum efficiency of the electroluminescence (EQE_EL_) test, this measurement was carried out at room temperature (297 K) in an N_2_-filled glovebox, the device was tested using a spectrometer (ZQE800, Nanjing Ouyi Optoelectronics Technology) and a photodetector (LST800), relying on the EQE-R-80. The device was placed on top of the integration sphere and only forward light emission can be collected. The devices were swept from zero bias to forward bias.


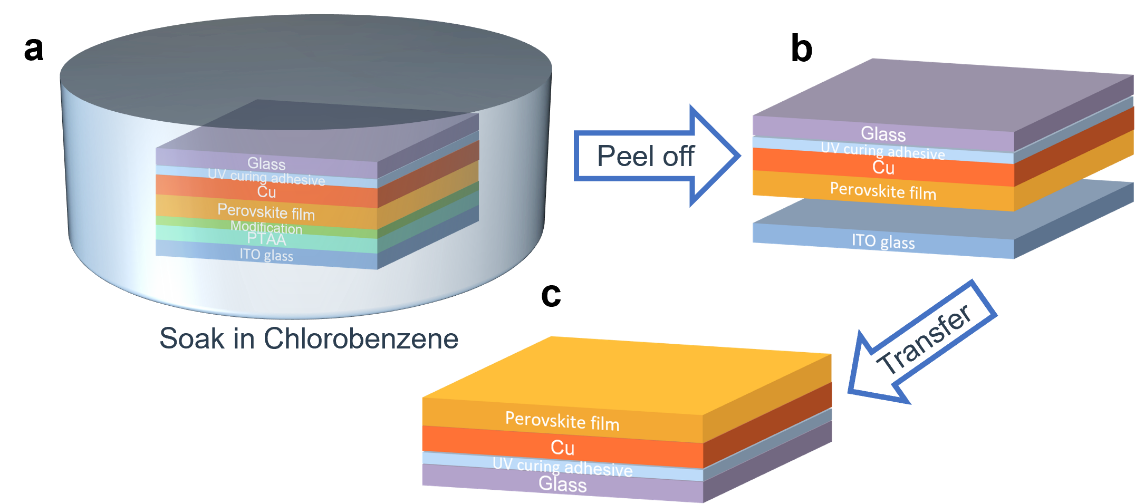


**Fig. S1.** Schematic diagram of the film uncovering process: a) After depositing the perovskite film on PTAA, vacuum vaporized a thick layer of copper (200 nm), adhered the glass cover plate to the copper by using UV curing adhesive, and immersed it in the chlorobenzene solvent. b) After an appropriate period (1-2 h) of immersing, the perovskite film which was separated from the substrate was promptly taken out from the chlorobenzene. c) Wait until the chlorobenzene on the wet film evaporates naturally, a series of characterization of the buried bottom interface can be carried out.

**
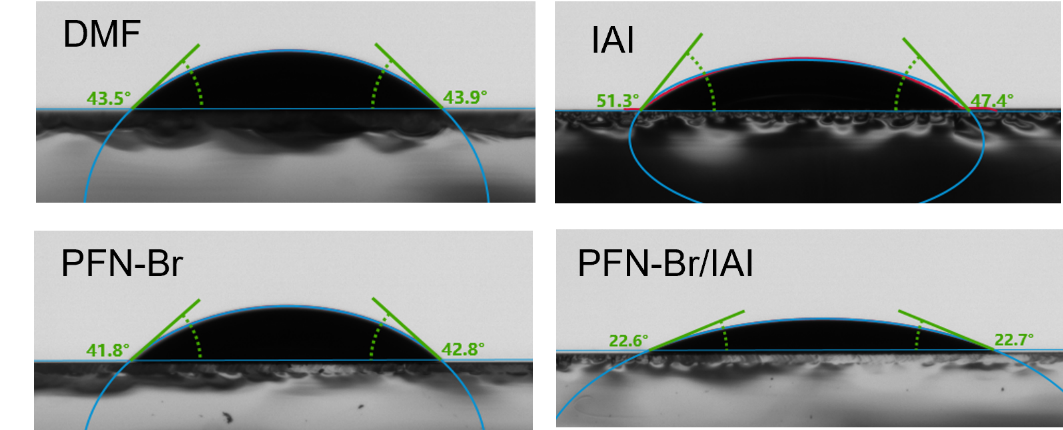
**

**Fig. S2.** The contact angle of perovskite precursor liquid on PTAA after DMF, IAI, PFN-Br, and PFN-Br/IAI treatments. Above are the images of precursor liquid staying on the surface for the 120th second.

**
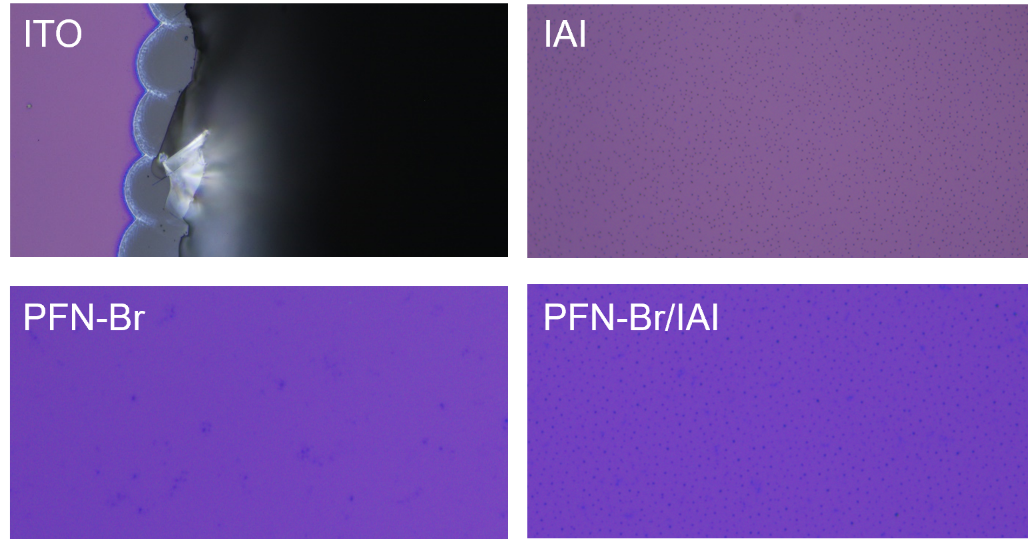
**

**Fig. S3.** Optical microscope images (1000×) on PTAA after treatments with DMF, IAI, PFN-Br and PFN-Br/IAI.

**
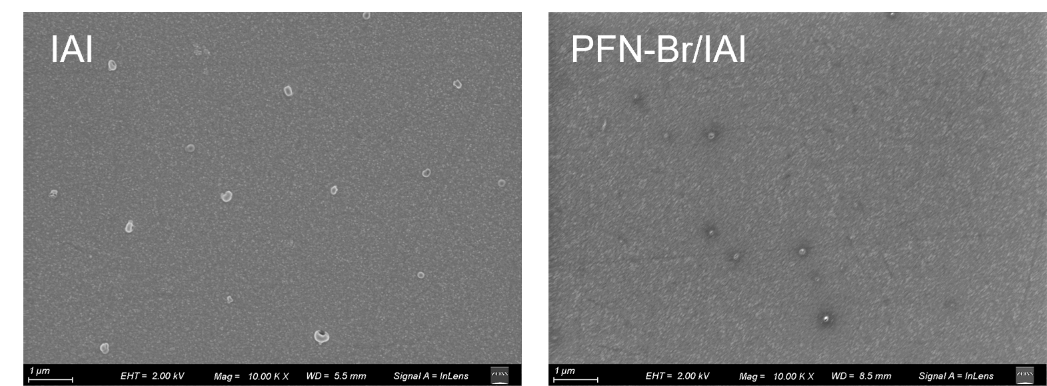
**

**Fig. S4.** Scanning electron microscopy (SEM) images on PTAA after treatments with IAI and PFN-Br/IAI.

**
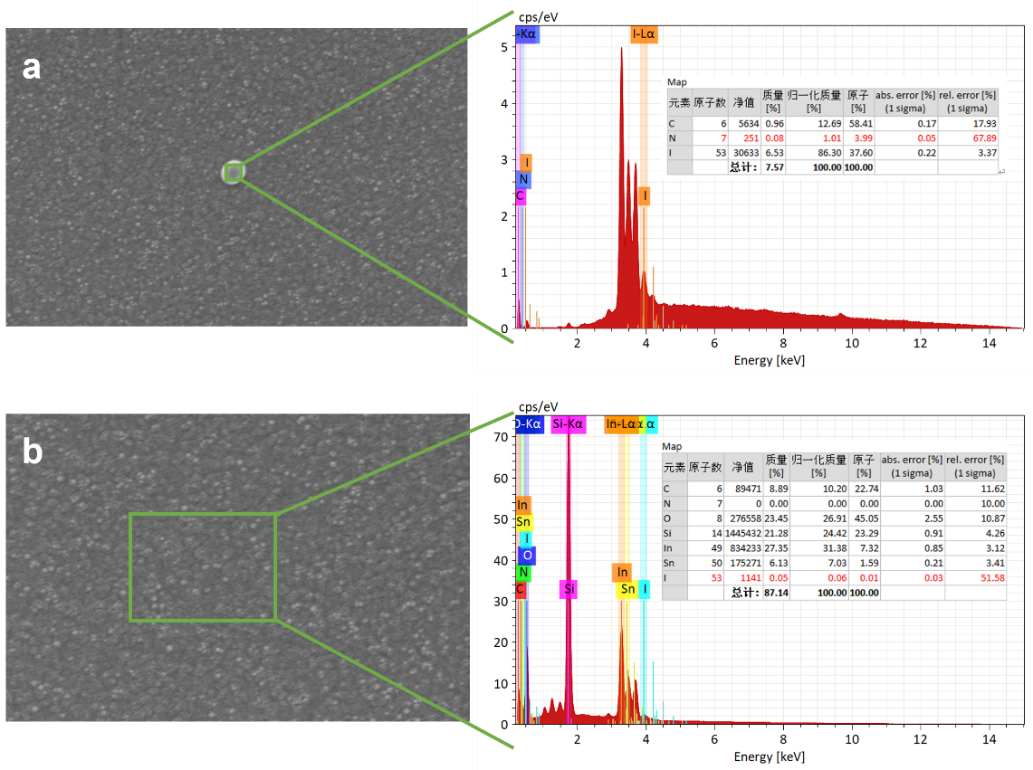
**

**Fig. S5.** X-ray energy spectrometer (EDS) mapping image analysis on PTAA/IAI substrate regarding a) island particles and b) blank regions, respectively.


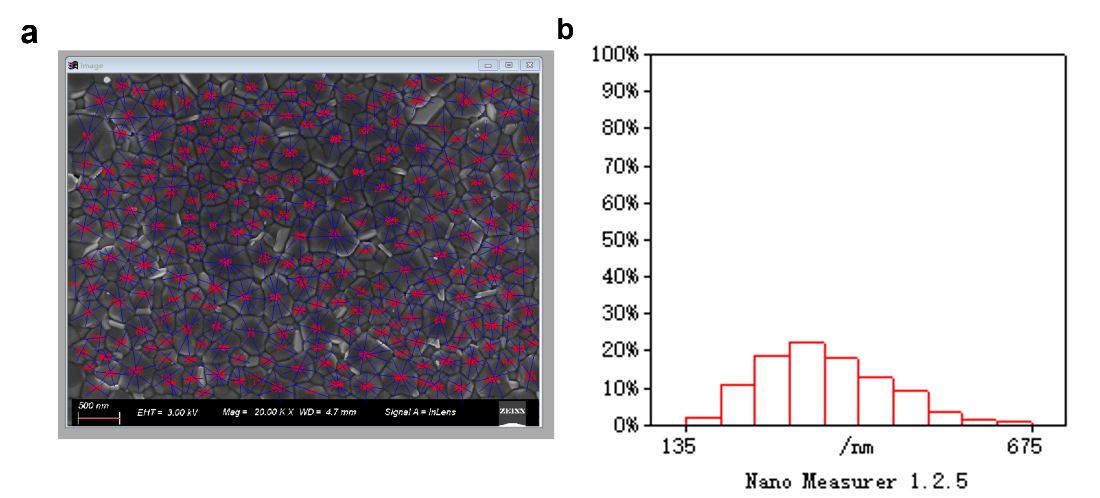


**Fig. S6.** SEM images of perovskite films and their associated a) grain size measurements and b) corresponding statistical distributions.


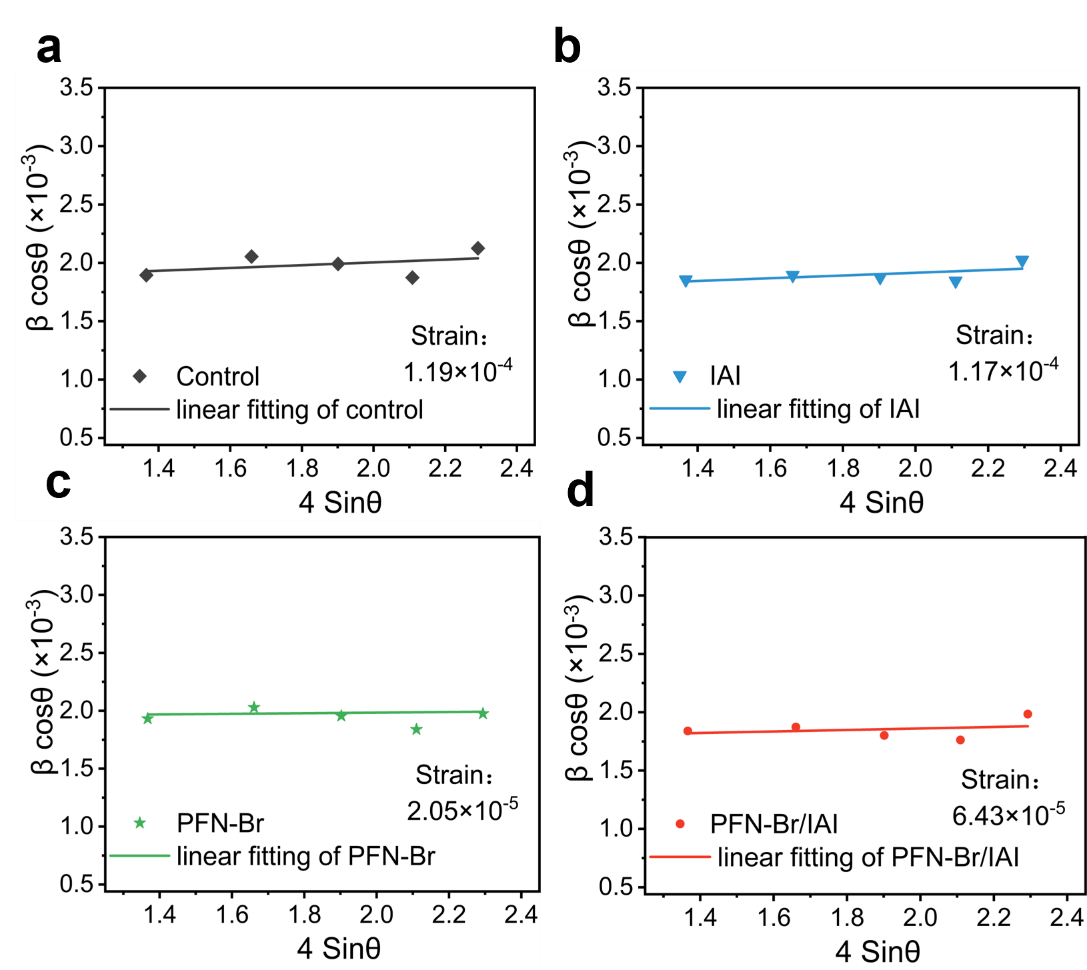


**Fig. S7.** Calculated and fitted residual strain of the perovskite crystal films deposited on different substrates by Williamson-Hall Plot, respectively.


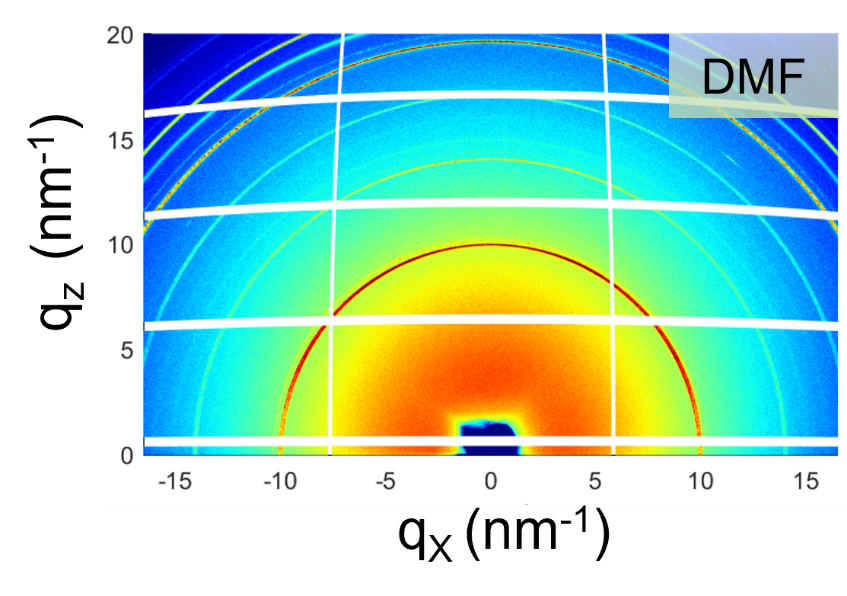


**Fig. S8.** Two-dimensional Grazing-incidence wide-angle X-ray scattering (GIWAXS) spectra of the bottoms of perovskite films bottoms deposited on DMF-treated PTAA layers, respectively (at 0.015° incidence angle).


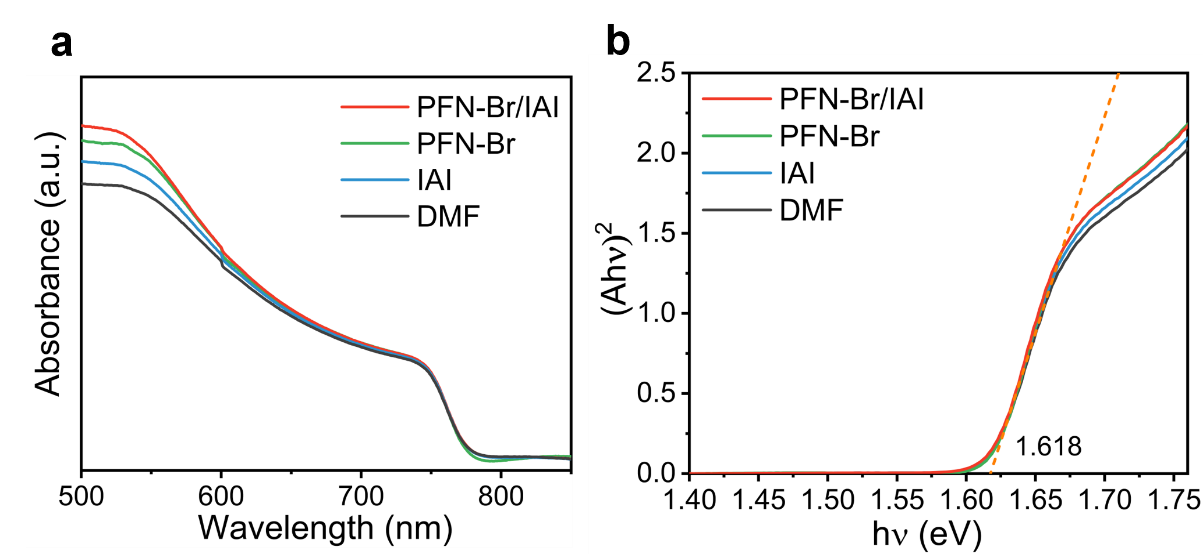


**Fig. S9.** a) UV-vis absorption spectra and b) Tauc plots of perovskite films deposited on ITO/PTAA substances treated with DMF, IAI, PFN-Br, and PFN-Br/IAI, respectively.


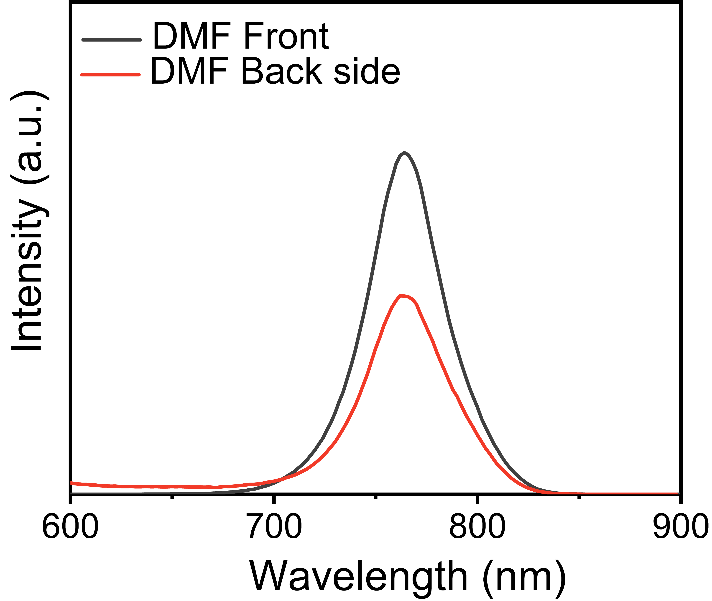


**Fig. S10.** PL spectra were collected from the top of the Glass/modified layer/perovskite films.


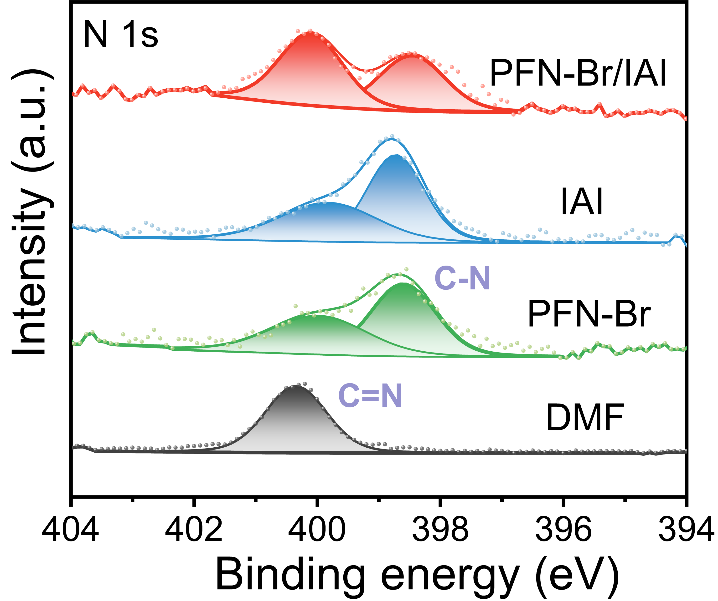


**Fig. S11.** X-ray photoelectron spectroscopy (XPS) analysis of N 1s concerning the bottom of perovskites deposited on different substrates. And C=N is from FA^+^ in the perovskite fractions and C-N is from N on IAI, PFN-Br.


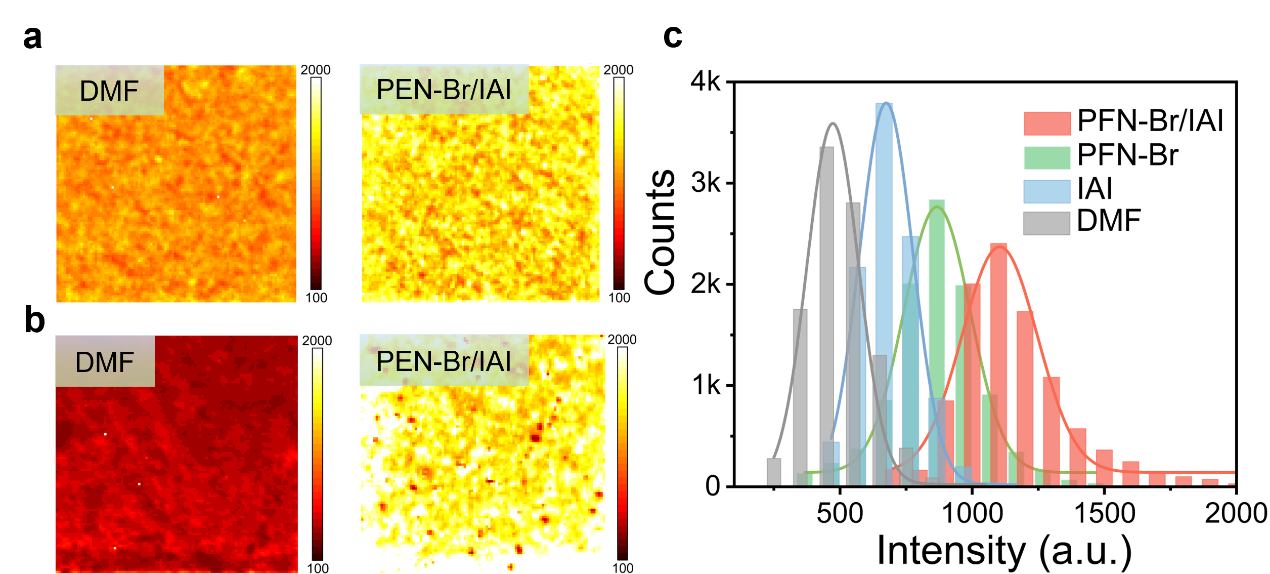


**Fig. S12.** a-b) The PL mapping images of the upper and buried interfaces of the control group (DMF) and the target group (PFN-Br/IAI) over the same scale; c)PL intensity distribution for confocal photoluminescence imaging (PL mapping) regarding the exposed bottom of perovskite films deposited on different substrates.

**
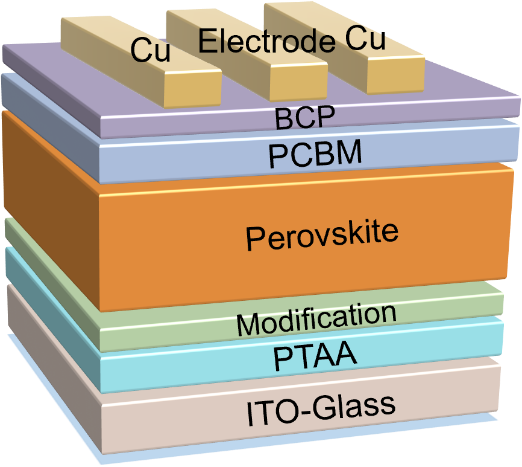
**

**Fig. S13.** Schematic structure of PTAA-based inverted devices.


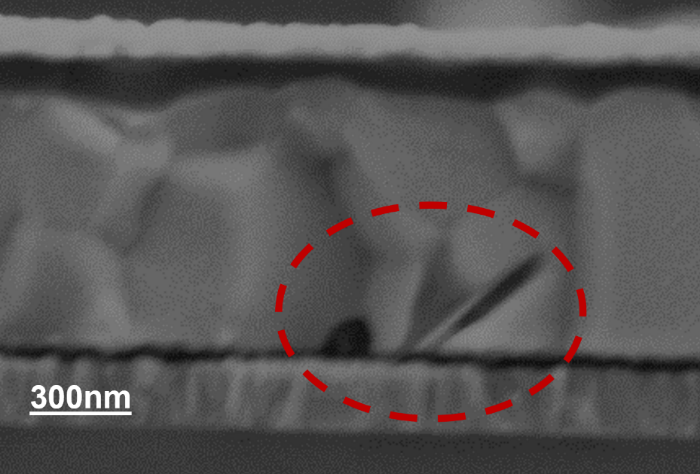


**Fig. S14.** Cross-sectional SEM image of the p-i-n device based on PTAA/DMF Rinse treatment with some pin-hole formation at the bottom and high grain boundary density.

**
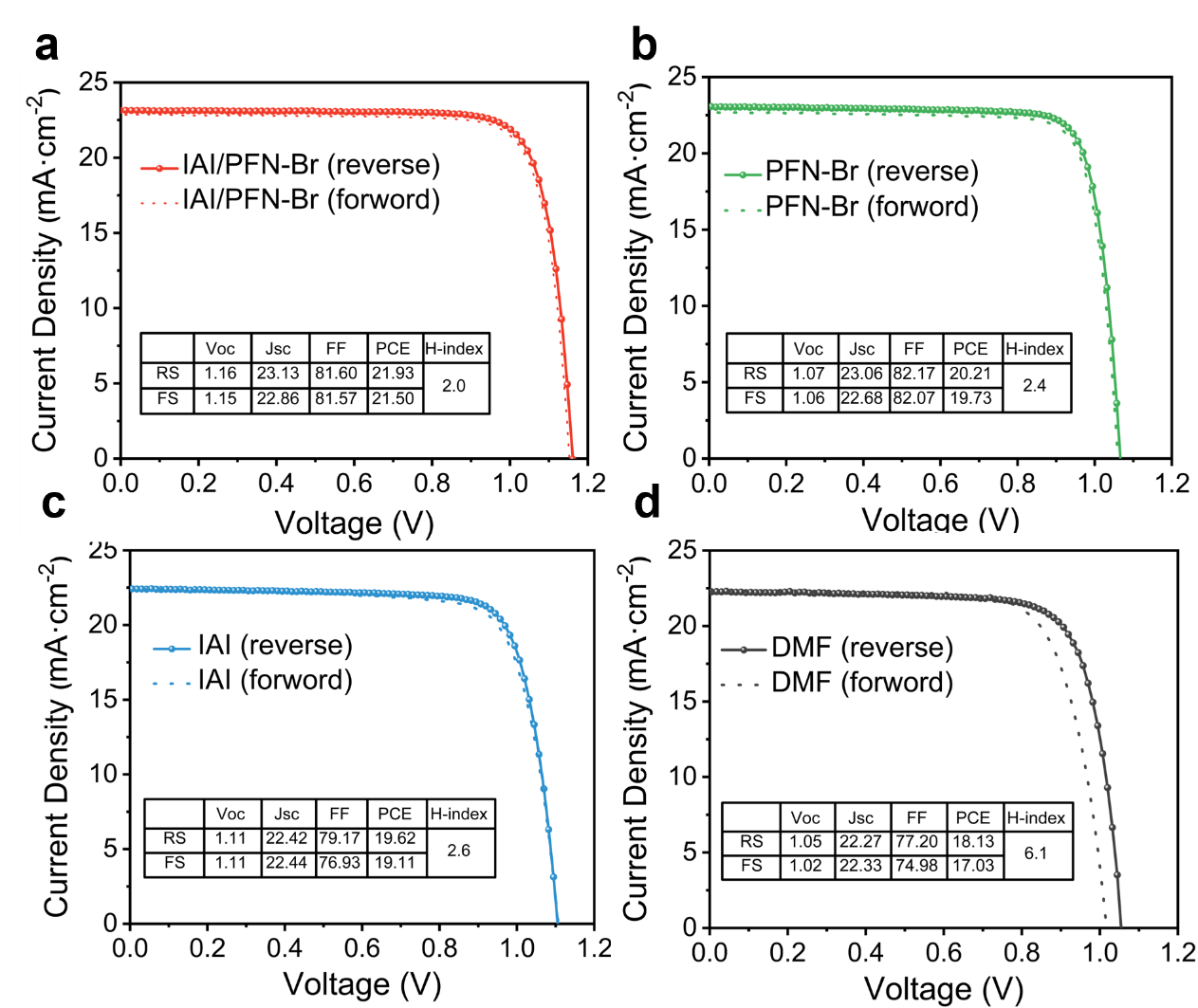
Fig. S15.** Forward/reverse scanning J-V curves and corresponding hysteresis factors (H-index) of PSCs based on different substrates under simulated standard AM 1.5 G illumination. Note: HI (hysteresis index) is calculated from H-index=(PCE_reverse_-PCE_forward_)/PC_Ereverse_


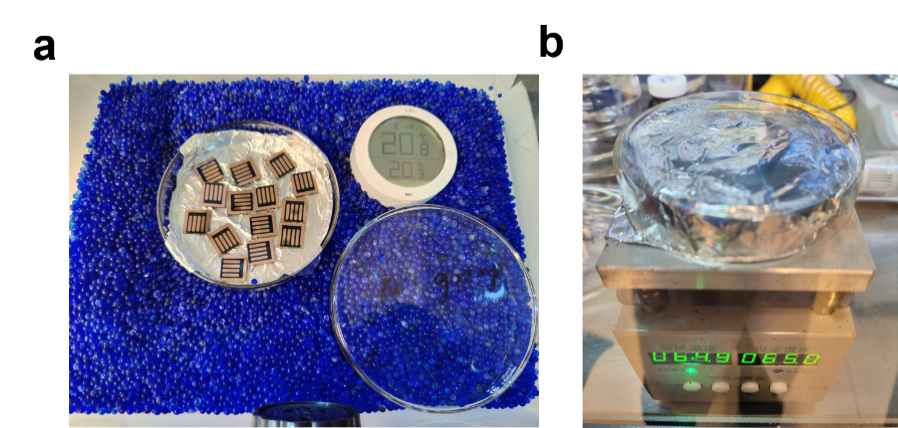


**Fig. S16.** a-b) Photographs of stability tracking tests of PSCs in air ambient (dark condition), under heating (dark condition) , respectively.

**
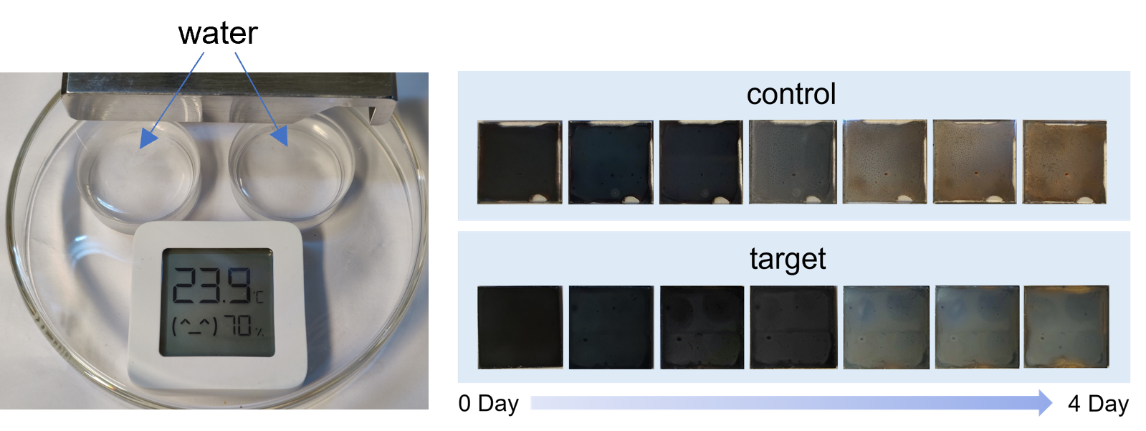
**

**Fig. S17.** The process of degradation of unencapsulated perovskite films (control: DMF, target: PFN-Br/IAI) was accelerated in air and high humidity (RH=70±10%). Notes: In an air environment, two exposed perovskite films and two small cups of water were covered with a glass petri dish with a heavy metal block pressed on top.

**Table S1.** Calculation of actual stresses in control and target perovskite thin films by grazing incidence X-ray diffraction spectroscopy (GIXRD).

| **Groups** | **2*θ*-sin^2^*Ψ*** | **2*θ_0_*** | ***σ* (MPa)** |
| --- | --- | --- | --- |
| DMF | 0.2742 | 31.75 | -112.21 |
| PFN-Br/IAI | 0.0921 | 31.73 | -39.78 |

**Table S2.** Carrier lifetime extracted from the bi-exponential fitted TRPL spectra.

| **Groups** | **τ_1_ (ns)** | **τ_2_ (ns)** | **A_1_** | **A_2_** | **τ_ave_ (ns)** | **R^2^** |
| --- | --- | --- | --- | --- | --- | --- |
| DMF | 9.48 | 645.46 | 0.24 | 0.76 | 642.44 | 0.996 |
| IAI | 12.96 | 1396.8 | 0.28 | 0.72 | 1391.86 | 0.988 |
| PFN-Br | 12.31 | 1783 | 0.19 | 0.80 | 1780.04 | 0.985 |
| PFN-Br/IAI | 15.35 | 2248.7 | 0.29 | 0.71 | 2242.56 | 0.985 |

**Table S3.** Trap filling limit voltage (V_TFL_) and corresponding defect density (N_t_) for HTL-only Devices

| **groups** | **V_TFL_ (V)** | **N_t_ (cm^-3^)** |
| --- | --- | --- |
| DMF | 0.601 | 5.99056E+15 |
| IAI | 0.476 | 4.7446E+15 |
| PFN | 0.415 | 4.13658E+15 |
| PFN-Br/IAI | 0.352 | 3.50861E+15 |

**Table S4.** Statistics of photovoltaic parameters of PSC based on DMF(control) and PFN-Br/IAI (target) treated PTAA on quadruple-cation perovskite (Rb_0.05_Cs_0.05_MA_0.05_FA_0.85_)Pb(I_0.95_Br_0.05_).

| **Groups** | **V_OC_ (V)** | **J_SC_ (mA/cm^2^)** | **FF (%)** | **PCE (%)** |
| --- | --- | --- | --- | --- |
| DMF | 1.10 | 24.68 | 79.24 | 21.47 |
| PFN-Br/IAI | 1.15 | 25.03 | 82.70 | 23.74 |

**Note S1.** Lattice Residual Strain Calculation Method.

To verify the stress-relieving effect of PFN-Br/IAI on perovskite, based on the XRD test results and theoretical derivation and fitting calculations, the change of perovskite lattice strain was visualized. The total half-peak width (FWHM) of the XRD diffraction peaks is determined by the grain size (D) and the micro stress ($\beta_{\text{ε}}$):

$\beta_{T}=\beta_{\text{ε}}$+ $\beta_{D}$ (1)

Combined with Schell's formula:

$D_{WHP}=\frac{K\lambda}{\beta_{D}cos\theta}$ (2)

Along with the relationship between $\beta_{\text{ε}}$ and the angle of the peak position (θ)：

$\beta_{\text{ε}}=4\varepsilon tan\theta$ (3)

Where 𝛽_𝑇_ is the half-peak full width of the Bragg diffraction peak, the instrumental contribution to the spread is eliminated here, the shape factor k is a constant value, λ (= 1.5406 Å) is the wavelength of the X-rays, D_WHP_ is the average grain size.

Putting equation (2) and (3) in equation (1),we get,

$\beta_{T}=\frac{k\lambda}{D_{\mathrm{WHP}}\cos\theta}+4\varepsilon\tan\theta$ (4)

To simplify, the residual lattice stress (ε) can be obtained as follows:

$\beta_{T}\cos\theta=\frac{k\lambda}{D_{\mathrm{WHP}}}+4\varepsilon\sin\theta$ (5)

With the above derivation, we calculated the residual lattice strain after annealing of perovskite films on different substrates.


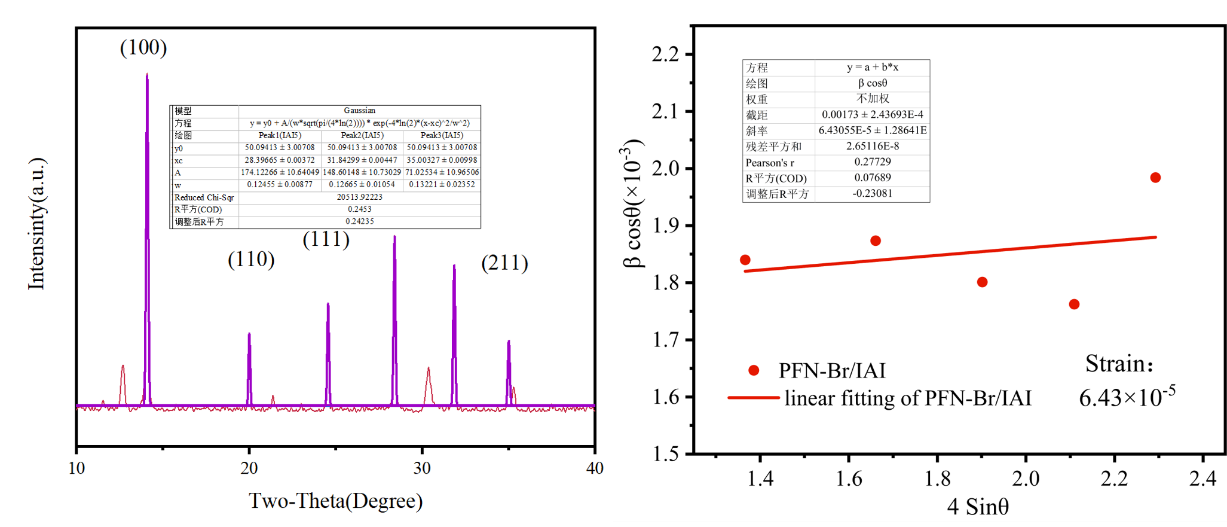


The process of calculating the residual strain of films on PTAA/PFN&IAI substrates based on XRD plots and Williamson-Hall plots is shown above.

**Note S2.** Fitting of average carrier lifetime (τ_avg_).

The τ_avg_ in the TRPL decay curve was fit using a double exponential function, and τ_avg_ can be calculated using the Equation below:

$\tau_{\mathrm{avg}}=\frac{A_{1}\tau_{1+}^{2}A_{2}\tau_{1}^{2}}{A_{1}\tau_{1}+A_{2}\tau_{2}}$ (6)

Where τ1 and τ2 are the fast decay component and slow decay component, respectively.

**Note S3.** Calculation of Defect Density (N_t_) in HTL-only Devices.

Space-charge limited current (SCLC) measurements were conducted on hole-only devices with a configuration of ITO/PTAA/modification layer/perovskite/Spiro-OMeTAD/Au. The N_t_ can be estimated according to the following equation:

$N_{t}=\frac{2\varepsilon_{0}\varepsilon V_{TFL}}{eL^{2}}$ (7)

Here, L is the thickness of the perovskite film (600 nm), e is the elementary charge, and $\varepsilon_{0}$、$\varepsilon$ represent the vacuum permittivity (8.8542 × 10^-12^ F/m) and the relative permittivity of perovskite (32.1), respectively.
